# Is Vaccination a Curse to the Human Race?

**Published:** 1892-11

**Authors:** C. A. Walters

**Affiliations:** Brookyln, N. Y.


The following two cases were read:

### IS VACCINATION A CURSE TO THE HUMAN RACE?

C. A. Walters, M. D., Brooklyn, N. Y.

"Who is he that will plead with me."—*Job* xiii, 19.

General attention has been called to this subject within the last few days throughout the United States and Canada by the newspapers publishing an account of the suffering and death of Miss Frances Asten, residing at 224 Eckford Street, Brooklyn, N. Y.

As her family physician, a short history of her case, together with several others due to the same cause, may be of interest to my brethren in the profession, and, I hope, be the means of arousing sufficient energy in the people to overthrow that outrageous practice — compulsory vaccination in our public schools.

On December 1st, 1885, by the request of Mr. Asten, I vaccinated his two children: Frances, aged ten years, and Edna, aged eight years. He informed me at the time that he was opposed to it, had no faith in it, and would not have either one vaccinated were it not for the fact that both girls had been ordered by the Board of Education not to come to school again until they had been vaccinated.

Five days after the operation Frances complained of a pain in her left side, in the region of the heart; the pain grew steadily worse and two days later she became paralyzed.

Three days later hyperæsthesia of sensory nerves manifested itself to such an extent that she was unable to move her limbs without suffering torture, and whenever she was touched she screamed with pain. At this stage of her case I called the late Professor Lilienthal in consultation, who reluctantly gave it as his opinion that the vaccine virus was the cause of her illness.

On December 13th, an abscess formed just over her left knee, and the pain became so intense that she was unable to sit on a chair or stand erect. As time passed, other abscesses appeared in various parts of the body, more especially within the abdomen, which, in spite of all treatment, involved the intestines, resulting in the escape of fecal matter from the several openings.

Professor Janeway, and others equally prominent in our profession, were called in for aid, but all remedies seemed powerless to touch her case. Even the *maximum* doses of various narcotics failed to ease her torment, her screams being heard a block away from her home.

She wasted away slowly, and from August, 1889, she was a helpless cripple. At her death on January 15th, 1892, she weighed forty-seven pounds. As a matter of justice I desire to mention that on the same day that Frances and her sister were inoculated I vaccinated ten others, none of whom had any unusual symptoms follow the operation. The virus was on ivory points and was from the New England Company.

I shall make no comments at present, simply contenting my self with the statement that Frances was in *robust* health at the time I vaccinated her.

On the 6th of January, 1887, the press of New York city and Brooklyn announced in large type:

"POISONED!!!

"TWO DEAD AND ONE DYING—HUSBAND AND SEVEN CHILDREN POISONED.

"Coroner Rooney, of Brooklyn, was notified to hold an inquest in the case of Joseph Mauri, aged twelve years, and Eugene, aged twenty months, who died yesterday from poisoning, at their residence, No. 111 Butler Street, Brooklyn. When the reporter called, he found Mr. Joseph Mauri, a druggist doing business at 447 Hicks Street, at the point of death, together with the remainder of his children, five of them, prostrated with the same symptoms. The wife and mother of the family is the only one who is not prostrated, and circumstances point to her as the guilty one."

The diagnosis was made by three physicians, viz., Dr. Raub, Dr. Elias H. Bartley, and Dr. William E. Griffiths, the two last being members of the Board of Health, and present in their official capacity. The symptoms noted on their first visit were:

"*Excessive* vomiting with *burning* in stomach, no fever nor purging, pains in the back and abdomen, with a livid, bluish, mottled condition of the skin, as sometimes noticed in cases of malignant scarlet fever." The dead were removed to the morgue and a *post-mortem* made, which proved very startling, to say the least. I again quote from the newspapers of the next day:

"VERY VIRULENT SMALL-POX.

"THE MAURI FAMILY ILLNESS NOT DUE TO POISON.

"The *post-mortem* made yesterday on the bodies of John and Eugene Mauri shows the illness of the family to be variola hemorrhagia, which is the so-called black small-pox, or small-pox of the most malignant type, and is not due to poisoning, as was supposed to be the case yesterday. Mrs. Mauri, when seen, could not account for the disease appearing in her family, except it was caused by their being vaccinated on last Thursday, December 30th, by order of the Board of Education. Dr. Raub, the family physician, persuaded Mr. Mauri (who was opposed to vaccination) to allow him to vaccinate the entire family. Mr. Mauri and the seven children were inoculated, but when Mrs. Mauri was requested to also submit to it she emphatically refused."

The father died the next day, making the fourth victim in this family.

---

The resolutions by Dr. Hitchcock were referred to a committee consisting of Drs. Hitchcock, A. R. Morgan, and Defriez.

